# Availability and use of essential medicines in China: manufacturing, supply, and prescribing in Shandong and Gansu provinces

**DOI:** 10.1186/1472-6963-10-211

**Published:** 2010-07-17

**Authors:** Wen Chen, Shenglan Tang, Jing Sun, Dennis Ross-Degnan, Anita K Wagner

**Affiliations:** 1 Department of Health Economics, School of Public Health, Fudan University and Key Laboratory of Public Health and Safety, Ministry of Education, 138 Yi Xue Yuan Road, Shanghai 200032, China; 2 TDR - UNICEF/UNDP/WORLD BANK/WHO Special Programme for Research and Training in Tropical Diseases, World Health Organization, 20 Avenue Appia, Geneva 1211, Switzerland; 3 International Health Group, Liverpool School of Tropical Medicine, Pembroke Place, Liverpool, L3 5QA, UK; 4 WHO Representative Office, 401 Diplomatic Office Building, 23 Dongzhimenwai Avenue, Beijing 100600, China; 5 Department of Population Medicine, Harvard Medical School and Harvard Pilgrim Health Care Institute, 133 Brookline Avenue, Boston, Massachusetts 02215, USA

## Abstract

**Background:**

The current health care reform in China launched in 2009 tackles the problem of access to appropriate medicines for its 1.3 billion people by focusing on providing essential medicines to all. To provide evidence for the reform process, we investigated the manufacturing, purchasing, and prescribing of essential medicines in two provinces.

**Methods:**

We conducted surveys in 2007 of all manufacturers (n = 253) and of 59 purposively selected retail and 63 hospital pharmacies in Shandong and Gansu provinces to assess production and supply of products on the 2004 National Essential Medicines List (NEML), as well as factors underlying decision making about production and supply. We also reviewed prescriptions (n = 5456) in health facilities to calculate standard indicators of appropriate medicines use.

**Results:**

Overall, manufacturers in Shandong and Gansu produced only 62% and 50%, respectively, of the essential medicines they were licensed to produce. Of a randomly selected 10% of NEML products, retail pharmacies stocked up to 60% of Western products. Median availability in hospital pharmacies ranged from 19% to 69%. Manufacturer and retail pharmacy managers based decisions on medicines production and stocking on economic considerations, while hospital pharmacy managers cited clinical need. Between 64% and 86% of prescriptions contained an essential medicine. However, overprescribing of antibiotics (34%-77% of prescriptions) and injectables (22%-61%) for adult non-infectious outpatient consultations was common.

**Conclusions:**

We found that manufacturers, retail pharmacies, and hospital pharmacies paid limited attention to China's 2004 NEML in their decisions to manufacture, purchase, and stock essential medicines. We also found that prescribing of essential medicines was frequently inappropriate. These results should inform strategies to improve affordable access to essential medicines under the current health care reform.

## Background

China spent 4.5% of Gross Domestic Product (GDP) on health care in 2007 [1]. Although total health care expenditures have been modest relative to GDP, pharmaceutical expenditures account for a significant proportion, averaging over 40% during the past two decades [1]. In contrast, the average percentage in OECD countries was around 15% [2].

Despite high pharmaceutical spending, China experiences substantial problems in access to medicines, due both to the lack of availability of essential medicines and to the high cost of and preference for branded products [3]. Perverse financial incentives to service providers lie at the core of these problems. A large proportion of hospital revenue comes from profits from pharmaceutical sales, often the most important source of income at county and lower level hospitals and health centers [4,5]. Service providers make greater profits on higher priced pharmaceuticals, since the mark-up rate is fixed by government regulation. Hence, Chinese doctors tend to overprescribe medicines, in particular expensive medicines, to maximize revenue generation for their institutions and bonus payments for themselves [5,6].

For over 30 years, the WHO has advocated an essential medicines list for member states [7]. Every two years since 1977, WHO has updated the WHO Model List of Essential Medicines of about 300 products, which countries are expected to adapt to their needs [8]. China's Ministry of Health developed its first National Essential Medicine List (NEML) in 1981, aiming to ensure the adequate supply, distribution, and rational use of essential medicines. The 2004 NEML consisted of 1260 Chinese herbal preparations and 773 chemical and biological medicines products. However, appropriate supporting policies and mechanisms needed for the NEML to achieve its intended objectives have been lacking in the areas of manufacturing, supply, reimbursement and use of essential medicines [3].

To guide the pharmaceutical sector, Chinese authorities have formulated a series of policies on pharmaceutical research and development, product approval, production, distribution, utilization, pricing, and insurance coverage [9]. Of these, price management and insurance coverage have been the two most important measures influencing availability and use of essential medicines. Controls on medicine pricing have been promulgated 27 times since 1997, but the measures have not had significant impact in reducing financial burden for service users. One reason is that manufacturers stop producing medicines that no longer yield targeted profits, and hospitals and doctors are not keen to use them for similar reasons [9-11].

Few studies have examined the availability and use of essential medicines in China, one of four key health sector components targeted in China's ambitious 2009 health system reform plan. In this paper, we provide evidence to answer the following research questions: To what extent are essential medicines produced by Chinese manufacturers and how available are these medicines in retail and hospital pharmacies? How frequently and appropriately are essential medicines prescribed in Chinese health facilities?

## Methods

We conducted the current study in Shandong and Gansu provinces, which are representative of the eastern (more developed) and western (less developed) regions of China, respectively. To understand availability of essential medicines, we conducted surveys in 2007 of manufacturers, hospitals, and retail pharmacies. To understand medicines use patterns, we reviewed a sample of prescriptions in selected hospitals. Table 1 contains an overview of the information collected from each data source. The study was funded by the WHO, which agreed to the use of these data for academic research.

**Table 1:** Data sources for manufacturer, pharmacy, and prescription surveys

Provinces (population, n)	Shandong (92 million)			Gansu (17 million)		
Manufacturers, n	217			36		

Hospital level of care	Primary	Secondary	Tertiary	Primary	Secondary	Tertiary

Study hospitals, n	15	17	8	7	11	5

Prescriptions surveyed, n	982	1687	800	414	1078	495

Prefecture GDP/capita	High	Middle	Low	High	Middle	Low

Study retail pharmacies at city-level, n						
With insurance contracts	2	2	2	2	2	2
Without insurance contracts	3	3	3	3	3	3

Study retail pharmacies at county level, n	5	5	5	5	5	4

### Manufacturer survey

For the purpose of this survey, all manufacturers in Shandong and Gansu provinces registered with the SFDA were requested to report to the SFDA the essential medicines which they were licensed to produce and those which they actually produced in 2004 and 2005. We did not obtain data on non-essential medicines production. In structured interviews, we also asked the chief executives of these manufacturers which factors most influenced their essential medicines production decisions.

### Health facility pharmacy survey

We selected representative, non-random samples of 10% of the primary, secondary, and tertiary care hospitals in each province. Hospital levels differ by technical capacity. Primary hospitals (known as urban community health care centers and rural township hospitals) deliver comprehensive primary care and limited inpatient care for common diseases. Secondary hospitals (known as urban district hospitals and rural county hospitals) are responsible for basic medical care, emergency care, and technical instruction to physicians in primary hospitals. Tertiary hospitals provide diagnosis and treatment for complex diseases and technical instruction to secondary hospitals.

In each facility, we reviewed 2006 pharmacy purchasing records to assess the percentage of essential medicines among all Western medicines purchased. We also assessed the availability in pharmacy stocks of selected essential medicines (see below). Using structured questionnaires, we interviewed pharmacy managers about their reasons for purchasing and stocking essential medicines.

### Retail pharmacy survey

Although hospital pharmacies dispense prescribed medicines for most outpatients, retail pharmacies, which are managed by licensed pharmacists, can also dispense OTC and prescription medicines. We divided all prefectural level cities in the two study provinces into those with high, middle, and low average GDP per capita. We randomly selected one study city to represent each socioeconomic group; in each city, we purposively selected two pharmacies contracted to dispense medicines to insured patients and three pharmacies without insurance contracts. We then purposively selected one middle GDP county (the administrative level below the prefectural level) in each study city; in each county, we selected five representative retail pharmacies. In total, we surveyed 59 retail pharmacies, 30 in Shandong and 29 in Gansu.

In study pharmacies, we assessed availability of a sample of essential medicines (see below). Study personnel visited each pharmacy to collect these data and to interview pharmacy managers about their rationale for purchasing and stocking essential medicines and about their understanding of the essential medicines concept.

### Selection of survey medicines

To assess availability of essential medicines in retail and hospital pharmacies, we randomly selected 40 Chinese medicines (107 unique dosage forms) and 77 Western medicines (98 unique dosage forms) from the 1260 Chinese and 773 Western medicines listed on the 2004 NEML. Since product strength is not indicated on the NEML, we included any available strength of the sample of Western medicines. Although the availability of Chinese herbal preparations improves access to essential medicines, especially in rural primary health care institutions, Western medicines are the treatment of choice for the majority of Chinese patients.

### Prescription review

Using clinic records, we reviewed outpatient prescriptions in each study hospital on a random day in 2006, following survey methods recommended by WHO for investigating prescribing in health facilities [12]. This survey sought to characterize the overall rate of prescribing of medicines from the NEML in routine adult outpatient care, as well as the rates of specific potentially inappropriate prescribing practices, including polypharmacy and overprescribing of antibiotics and injections. We systematically selected 100 prescriptions in each facility. If fewer than 100 prescriptions were issued, we included all prescriptions issued on the study day. To assess prescribing of Western medicines, we excluded herbal products. To examine potential overprescribing of antibiotics and injections in routine adult outpatient care, we excluded prescriptions for children, adult emergency care, and adult cases treated in hospital infectious disease clinics.

### Data analysis

Data were analyzed using SPSS version 13.0. We summarize data on production as the percentage of licensed essential medicines manufactured and identify the 10 most frequently manufactured essential medicine products. We summarize the supply of essential medicines in pharmacies by category (Chinese and Western medicines on the NEML). We list the factors most frequently cited by enterprise chief executives and pharmacy managers when deciding on producing or procuring essential medicines, respectively. We also describe the frequency of inappropriate prescribing using standard indicators of quality use of medicines [13].

## Results

### Manufacturing of essential medicines

Shandong manufacturers averaged 20 essential medicine product licenses in 2005, with about 60% of licensed products actually produced (Table 2). In Gansu, manufacturers averaged 41 licenses but only 50% of the products were manufactured in 2005. The proportion of essential medicines produced was not associated with manufacturer sales volumes. Among manufacturers that failed to report sale volumes, the proportion of licensed products actually produced was very low (1.1%).

**Table 2:** Essential medicines production as percentage of licenses held by manufacturers in Shandong and Gansu provinces in 2005, by manufacturer sales volume

Annual sales volume, RMB*	Shandong			Gansu		
	
	Manufacturers, n	Essential medicines licenses held, n	Licensed essential medicines produced, n (%)	Manufacturers, n	Essential medicines licenses held, n	Licensed essential medicines produced, n (%)
Less than 10 million	54	440	271 (62)	13	262	102 (39)
10-30 million	51	778	463 (60)	12	610	306 (50)
30-100 million	48	1117	665 (60)	5	455	273 (60)
100-500 million	35	1292	872 (67)	3	136	57 (42)
More than 500 million	12	622	399 (64)	0	0	0
Unknown	17	91	1 (1)	3	26	0
Total	217	4340	2671 (62)	36	1489	737 (50)

* On Jan 1, 2007, near the time of the survey, the conversion rate was RMB 7.80 to $US 1.00.

In Shandong and Gansu provinces, respectively, the five factors mentioned by manufacturers as most influential on their production decisions were market demand (81%, 94%), production cost (79%, 89%), price (65%, 50%), market share (54%, 56%), and profit margin (54%, 53%). Whether or not the medicines were listed as reimbursable by the social health insurance (32%, 25%) or listed in the NEML (20%, 25%) were less important.

At least one manufacturer produced 579 (28.5%) and 230 (11.3%) of the essential medicine products on the 2004 NEML in Shandong and Gansu provinces, respectively. The most frequently produced product was glucose injection (produced by 117 manufacturers in Shandong and 18 in Gansu). In Shandong, of the ten most frequently manufactured products, eight were Western medicines and six of those were injections. Paracetamol tablets, a widely used pain reliever for adults and children, was among the ten most frequently licensed products (by 36 manufacturers), but not among the ten most frequently manufactured ones (produced by ten manufacturers). Among the ten most frequently manufactured products in Gansu, glucose injection was the only Western medicine (Table 3).

**Table 3:** Top 10 essential medicines products manufactured in 2005

Shandong			Gansu		
**Medicine (name, dosage form)**	**Manufacturers with license, n**	**Manufacturers producing, n**	**Medicine (name, dosage form)**	**Manufacturers with license, n**	**Manufacturers producing, n**

Glucose injection	177	117	Glucose injection	20	18

Sodium chloride injection	96	64	Xiaoyao pills	22	17

Glucose and sodium chloride injection	88	59	Liu Wei Di Huang pills	20	17

Banlangen granule	31	25	Bao He pills	17	14

Vitamin C injection	57	23	Gui Fu Di Huang pills	17	13

Metronidazole tablet	36	22	Bu Zhong Yi Qi pills	21	12

Norfloxacin capsule	37	21	Guipi pills	16	12

Ribavirin injection	35	21	Cen Su pills	15	12

Liu Wei Di Huang pills	30	21	Fuzi Lizhong pills	17	11

Metronidazole injection	29	20	Huang Lian Shang Qing pills	16	11

### Supply of essential medicines

#### Retail pharmacies

Of the 140 Western essential medicine products randomly sampled from the NEML, 49 (35%) and 69 (49%) were not available in any investigated retail pharmacy in Shandong and Gansu, respectively. Among the 41 NEML products not found in either province, 21 were injections, including diagnostics like technetium and iodohippurate, as well as products like potassium phosphate which require administration in hospital settings. However, essential medicines like salbutamol and sodium valproate syrups, used to treat asthma and epilepsy in children, respectively, were also not available. About two-thirds of the pharmacies stocked only 17% and 12% of the selected essential Western medicines in Shandong and Gansu, respectively.

Of 107 essential traditional Chinese medicines, 7 (7%) and 19 (18%) products were not for sale in Shandong and Gansu provinces, respectively, including five which were not available in either province. About two-thirds of the pharmacies stored 45% and 46% of Chinese products in Shandong and Gansu, respectively.

Pharmacy managers in Shandong and Gansu reported that the top four factors determining their procurement decisions were market demand (90%, 100% respectively), price (90%, 83%), profit margins (73%, 45%) and market share (53%, 45%). Whether or not medicines are reimbursable by the social health insurance (33%, 34%) or listed in the NEML (10%, 21%) were again less important. In addition, more than 40% of pharmacy managers in each province did not know the NEML and 60% did not consider it in purchasing decisions.

#### Hospital pharmacies

Essential medicine products constituted 67% (standard deviation (SD) 27%), 72% (SD 22%), and 80% (SD 10%) of the Western medicine products purchased in 2006 by primary, secondary, and tertiary care hospital pharmacies in Shandong, respectively. Of the random sample of essential medicines products, pharmacies in Shandong primary care hospitals supplied a median of 26% and tertiary care hospitals 69% of the Western medicines on the NEML; in Gansu, these figures were lower at 19% and 38% respectively (Table 4). However, supply in Shandong varied widely. One hospital pharmacy had less than 20% of the sampled Western essential medicines, while another had more than 80%. Regardless of hospital level, half of the pharmacies carried about 25% of the Chinese NEML products. In Gansu province, no hospital pharmacy stocked more than 60% of Western essential medicines. Six NEML products were not available in any surveyed hospital pharmacy in both provinces (pipotiazine injection, capreomycin injection powder, ritonavir oral liquid, compound salvia miltiorrhiza pills, niuhuang shangqing capsule, and buzhong yiqi decoction).

**Table 4:** Availability (median percentage, 25^th^, 75^th ^percentiles) of selected essential medicines in the sample of hospital pharmacies

	Shandong		Gansu	
**Hospital Level of Care**	**NEML-Chinese**	**NEML-Western**	**NEML-Chinese**	**NEML-Western**

Primary	25%	26%	33%	19%
	(21%, 36%)	(23%, 30%)	(17%, 33%)	(16%, 20%)

Secondary	23%	47%	29%	30%
	(20%, 33%)	(45%, 51%)	(22%, 34%)	(24%, 34%)

Tertiary	23%	69%	23%	38%
	(21%, 29%)	(61%, 74%)	(22%, 24%)	(37%, 43%)

The most frequent reason for not purchasing the selected essential medicines across hospital levels was lack of clinical use for the products (in Shandong 64%, 58%, and 52% and in Gansu 62%, 69%, and 70% of primary, secondary, and tertiary hospitals, respectively), followed by availability of clinical alternatives (Shandong: 31%, 32%, 41%; Gansu: 28%, 28%, 22%).

### Prescribing of essential medicines

Overall, as shown in Table 5, the average number of medicines prescribed per patient was lower in Shandong province and decreased by hospital level (an average of 3.2 medicines in primary, 2.5 in secondary, and 2.0 in tertiary level hospitals in Shandong, compared to 5.3, 3.9, and 2.6 in Gansu). However, despite prescribing fewer medicines, the costs per prescription (and thus cost per medicine) increased substantially by hospital level.

**Table 5:** Indicators (mean ± SD) of outpatient medicines prescribing for routine adult outpatient consultations*

	Shandong			Gansu		
**Hospital level (number of Rx)**	**Primary**	**Secondary**	**Tertiary**	**Primary**	**Secondary**	**Tertiary**
	**(982)**	**(1687)**	**(800)**	**(414)**	**(1078)**	**(495)**

Average number of medicines/Rx	3.2 ± 0.7	2.5 ± 0.4	2.0 ± 0.3	5.3 ± 2.0	3.9 ± 1.1	2.6 ± 0.5

Average cost/Rx (RMB) **	38.6 ± 19.3	78.7 ± 27.5	101.3 ± 34.1	34.4 ± 28.3	48.2 ± 23.8	76.3 ± 16.0

% Rx with EM	72.5 ± 30.3	73.3 ± 19.3	63.8 ± 15.5	85.6 ± 25.3	78.1 ± 15.0	70.0 ± 13.1

% Rx with reimbursable medicines	76.4 ± 33.2	78.0 ± 17.0	79.3 ± 6.4	83.7 ± 26.3	77.4 ± 14.7	80.2 ± 12.7

% Rx with antibiotics	53.9 ± 9.1	45.9 ± 11.9	33.6 ± 14.0	77.2 ± 18.5	59.6 ± 8.7	40.7 ± 6.0

% Rx with injections	41.7 ± 11.2	31.8 ± 16.3	27.4 ± 16.3	61.1 ± 28.5	36.6 ± 14.0	22.3 ± 9.4

Rx = prescription

* Pediatric, adult emergency care, and infectious disease clinic consultations were excluded

** On Jan 1, 2007, near the time of the survey, the conversion rate was RMB 7.80 to USD 1.00.

While the percentages of essential medicines prescribed ranged from 64% in tertiary facilities in Shandong to 86% in primary hospitals in Gansu, large proportions of prescriptions contained an antibiotic (between 34% in tertiary care hospitals in Shandong and 77% in primary care hospitals in Gansu) or an injection (from 22% in tertiary care to 61% in primary care hospitals in Gansu). The percentages of prescriptions containing an antibiotic or injection were higher at lower levels of hospital care in both provinces.

## Discussion

Our analyses showed that manufacturers in Shandong and Gansu provinces did not produce at least 40% of the products on the 2004 NEML for which they held production licenses, with their production decisions determined primarily by economic considerations. Many essential medicines are not perceived as profitable because of low demand, as well as price and mark-up controls.

Most retail pharmacies stocked less than 20% and most hospital pharmacies between 20% and 74% of a random sample of Western products on the NEML, depending on the province and hospital level of care. While retail pharmacies cited primarily economic reasons for their purchase decisions, hospital pharmacies most commonly cite lack of clinical utilization as the reason for not stocking medicines on the NEML. However, clinical and economic motivations are closely related. Pharmacies tend to stock what is prescribed, and prescribing is motivated in part by financial incentives. Clinicians in hospitals favor prescribing of higher cost medicines not subject to price controls because they generate greater revenues. This puts added cost burden on patients, especially for those without insurance who pay for all medicines out of pocket and also on both urban and rural health insurance funds.

A fairly high percentage of the medicines prescribed in adult hospital outpatient encounters were included on the 2004 NEML, which is not surprising given that the list contained 773 Western essential medicines compared to the 300 on the WHO Model Essential Medicines List. Antibiotics and injections were very commonly prescribed. WHO estimates that guideline-based care would result in rates of antibiotic use in routine outpatient care of 20% or less and rates of injection use of 5% or less [12]. A recent WHO global review reported that the median rates of outpatient antibiotic and injection prescribing were 46% and 19%, respectively, for all published studies conducted between 2004 and 2006 [14]. These compare with the observed rates of antibiotic prescribing of 45% in Shandong and 59% in Gansu, and rates of injection prescribing of 34% and 38% in the two provinces, respectively. Thus, overprescribing of antibiotics and injections in these two provinces is particularly inappropriate, in light of global standards.

Our study has several limitations. We did not collect data on non-essential products manufactured and thus we cannot assess the proportion of essential medicines manufactured among all medicines produced. We assessed supply of a sample of NEML medicines, rather than all NEML products. Although we randomly sampled NEML medicines, it is possible that this sample is not representative of all essential medicines. In addition, since the NEML does not specify whether medicines are appropriate for inpatient or outpatient care, some of the NEML medicines may not be expected to be stocked in retail pharmacies. Lastly, because we did not have diagnostic information related to individual prescriptions, we could not assess appropriateness of prescribing in relationship to the condition treated.

These limitations not withstanding, the present data illustrate key challenges faced by the Chinese health care system. First, there are competing interests between the pharmaceutical industry's profit orientation and the government's objective of securing access to affordable essential medicines for the public. Over the past three decades, provincial and municipal governments have promoted the pharmaceutical industry as a pillar for economic growth and job creation [9,15], without emphasizing its responsibility in helping to secure access to essential medicines.

Second, the current medicines pricing system has failed to stimulate competition in the production of essential medicines. The pricing authority strictly controls the price of generics, while allowing higher prices for branded generics and much higher prices for originator products. To avoid price controls, manufacturers have shifted registration and marketing to branded generics. The data from Shandong province, where the pharmaceutical industry is an important component of the economy [16], show that manufacturers give priority to Western injectable products, among the more expensive products on the NEML.

Third, hospitals and doctors have no incentives to use relatively inexpensive generic essential medicines [9,11]. Since Government funding only accounts for about 10% of hospital funding [17], hospitals and health care providers have relied on out of pocket payments by individual patients or health insurance reimbursement. Health facilities generate greater profits through prescribing of medicines with high markups not subject to price control. The more medicines doctors prescribe, the higher the income hospitals and doctors receive [5]. Such perverse incentives have been a major obstacle in promoting rational use of medicines.

Consistent with other studies [18,19], we find that essential medicines constitute a reasonably high proportion of the medicines prescribed in hospitals. However, some prescribing of essential medicines can be inappropriate, such as prescribing injections for common conditions which can be safely treated with oral medicines or antibiotics for non-bacterial conditions in which they are not indicated. In addition to the economic incentives to overprescribe expensive medicines, a lack of knowledge among patients about essential medicines and the absence of effective training on appropriate use of medicines for health care professionals likely contribute to high levels of inappropriate use.

The Chinese Government has embarked upon major changes to overcome these challenges [20]. In August 2009, the Ministry of Health issued a new National Essential Medicines List for primary health care institutions, consisting of 205 western generic medicines and 102 Chinese herbal preparations) [21]. By 2012, all primary health care institutions with government subsidies in urban and rural areas will be required to stock and dispense these essential medicines with zero mark up. A maximum retail price will be set by the National Development and Reform Commission for each essential medicine and medicines with the same ingredient will have the same price, no matter whether the product is the originator, a branded generic, or a non-branded generic. All essential medicines will be covered by both urban and rural insurance schemes, and these medicines will be reimbursed at higher rates. Given the problems in the supply and use of the much larger 2004 NEML observed in this study, it will be important for the Government, insurance schemes, and health care institutions to establish policies and incentives that facilitate the use of the new NEML in manufacturing, purchasing, and prescribing decisions at each level of the health care system.

## Conclusions

In conclusion, we found that manufacturers, retail pharmacies, and hospital pharmacies paid limited attention to China's 2004 NEML in their decisions to manufacture, purchase, and stock essential medicines. We also found that prescribing of essential medicines was frequently inappropriate. These results should inform strategies to improve affordable access to essential medicines under the current health care reform.

## Competing interests

The authors declare that they have no competing interests. The WHO funded the study and agreed to use the data for academic research. The sponsor had no influence on the study design, collection, analysis, and interpretation of the data and the writing of the manuscript.

## Authors' contributions

WC led the study. He designed the study, led the data collection, analysis, and interpretation and wrote the first draft of the manuscript. ST contributed to the study design, provided input into the data analysis and interpretation, and helped the writing of the first draft manuscript. JS also contributed to the study design, data collection, and manuscript writing. AW and DR-D provided input into data analysis and interpretation and co-wrote the manuscript. All authors read and approved the final manuscript.

## Pre-publication history

The pre-publication history for this paper can be accessed here:

http://www.biomedcentral.com/1472-6963/10/211/prepub

## References

[B1] China National Health Economics Institute China National Health Accounts Report 2008 Beijing

[B2] Jacobzone S Pharmaceutical policies in OECD countries: Reconciling social and industrial goals. Paris Labour Market and Social Policy 2004 Occasional Papers No. 40

[B3] Ye L Chen W Ying X Liu B Hu S The problems and causes of essential medicines production, distribution and use Chinese Health Resources 2008 11 51 53

[B4] Yip W Eggleston K Addressing government and market failures with payment incentives: hospital reimbursement reform in Hainan, China Social Science & Medicine 2004 58 267 277 10.1016/s0277-9536(03)00010-8 14604613

[B5] Eggleston K Li L Meng Q Lindelow M Wagstaff A Health service delivery in China: A literature review Health Economics 2008 17 149 165 10.1002/hec.1306 17880024

[B6] Zhan S Tang S Guo Y Bloom G Drug prescribing in rural health facilities in China: Implication for service quality and cost Tropical Doctor 1998 28 42 48 9481197 10.1177/004947559802800112

[B7] Laing R Waning B Gray A Ford N Hoen E 25 years of the WHO essential medicines lists: progress and challenges Lancet 2003 361 1723 1729 10.1016/S0140-6736(03)13375-2 12767751

[B8] WHO Essential Medicines: WHO model lists of essential medicines http://www.who.int/medicines/publications/essentialmedicines/en/

[B9] Sun Q Santoro MA Meng QY Liu C Eggleston K Pharmaceutical policy in China Health Affairs 2008 27 1042 1050 10.1377/hlthaff.27.4.1042 18607039

[B10] Meng Q Cheng G Silver L Sun X Rehnberg C Tomson G The impact of China's retail drug price control policy on hospital expenditures: a case study in two Shandong hospitals Health Policy and Planning 2005 20 185 196 10.1093/heapol/czi018 15840634

[B11] Chen Y Schweitzer SO Issues in drug pricing, reimbursement, and access in China with references to other Asia-Pacific region Value in Health 2008 11 suppl 1 S124 129 10.1111/j.1524-4733.2008.00376.x 18387056

[B12] INRUD and WHO Action Programme on Essential Drugs How to investigate drug use in health facilities: selected drug use indicators 1993 World Health Organization, Geneva DAP Research Series N°7. WHO/DAP/93.1

[B13] WHO Using indicators to measure country pharmaceutical situation: Fact book on WHO level I and level II monitoring indicators (WHO/TCM/2006.2) 2006 World Health Organization, Geneva

[B14] WHO Essential Medicines and Pharmaceutical Policies Holloway K, Ivanovska V, Vialle-Valentin C, Johnson A, Lewis S, Wagner A, Ross-Degnan D Medicines use in primary care in developing and transitional countries: Fact Book summarizing results from studies reported between 1990 and 2006 2009 Geneva; World Health Organization WHO/EMP/MAR/2009.3

[B15] Han P China's growing biomedical industry Biologicals 2009 37 169 172 10.1016/j.biologicals.2009.02.010 19427231

[B16] Shandong Provincial Government Shandong Bio-industry Development Plan (2008~2012) http://www.shandong.gov.cn/art/2008/5/22/art_956_1360.html

[B17] Hu S Tang S Liu Y Zhao Y Escobar M-L de Ferranti D Reform of how health care is paid for in China: challenges and opportunities Lancet 2008 372 1846 1853 10.1016/S0140-6736(08)61368-9 18930520

[B18] Dong H Bogg L Rehnberg C Diwan V Association between health insurance and antibiotics prescribing in four counties in rural China Health Policy 1999 48 29 45 10.1016/S0168-8510(99)00026-3 10539584

[B19] Chen H Han L Yao X Ji B Analysis of the frequency and rationality of use of antibiotics in 571 patients China Pharmacy 2003 14 159 161

[B20] Chinese Communist Party Central Committee and the State Council Opinions on deepening health sector reform http://www.gov.cn/jrzg/2009-04/06/content_1278721.htm

[B21] Chinese Ministry of Health National Essential Medicines List for Primary Health Institutions http://www.gov.cn/gzdt/2009-08/18/content_1395524.htm

